# A Vulnerability Analysis for the Management of and Response to the COVID-19 Epidemic in the Second Most Populous State in Brazil

**DOI:** 10.3389/fpubh.2021.586670

**Published:** 2021-04-13

**Authors:** Igor Silva Campos, Vinícius Ferreira Aratani, Karina Baltor Cabral, Jean Ezequiel Limongi, Stefan Vilges de Oliveira

**Affiliations:** ^1^Undergraduate Medical, Faculty of Medicine, Federal University of Uberlândia, Uberlândia, Brazil; ^2^Undergraduate Course in Collective Health, Institute of Geography, Federal University of Uberlândia, Uberlândia, Brazil; ^3^Department of Collective Health, Faculty of Medicine, Federal University of Uberlândia, Uberlândia, Brazil

**Keywords:** COVID-19, social vulnerability, disease outbreaks, epidemics, policy formulation, health policy

## Abstract

The COVID-19 pandemic has the potential to affect all individuals, however in a heterogeneous way. In this sense, identifying specificities of each location is essential to minimize the damage caused by the disease. Therefore, the aim of this research was to assess the vulnerability of 853 municipalities in the second most populous state in Brazil, Minas Gerais (MG), in order to direct public policies. An epidemiological study was carried out based on Multi-Criteria Decision Analysis (MCDA) using indicators with some relation to the process of illness and death caused by COVID-19. The indicators were selected by a literature search and categorized into: demographic, social, economic, health infrastructure, population at risk and epidemiological. The variables were collected in Brazilian government databases at the municipal level and evaluated according to MCDA, through the Program to Support Decision Making based on Indicators (PRADIN). Based on this approach, the study performed simulations by category of indicators and a general simulation that allowed to divide the municipalities into groups of 1–5, with 1 being the least vulnerable and 5 being the most vulnerable. The groupings of municipalities were exposed in their respective mesoregions of MG in a thematic map, using the software Tabwin 32. The results revealed that the mesoregion of Norte de Minas stands out with more than 40% of its municipalities belonging to group 5, according to economic, social and health infrastructure indicators. Similarly, the Jequitinhonha mesoregion exhibited almost 60% of the municipalities in this group for economic and health infrastructure indicators. For demographic and epidemiological criteria, the Metropolitana de Belo Horizonte was the most vulnerable mesoregion, with 42.9 and 26.7% of the municipalities in group 5, respectively. Considering the presence of a population at risk, Zona da Mata reported 42.3% of the municipalities in the most vulnerable group. In the joint analysis of data, the Jequitinhonha, Vale do Mucuri and Vale do Rio Doce mesoregions were the most vulnerable in the state of MG. Thus, through the outlined profile, the present study proved how socioeconomic diversity affects the vulnerability of the municipalities to face COVID-19 outbreak, highlighting the need for interventions directed to each reality.

## Introduction

In late December 2019, hospitals in Wuhan, China, identified numerous patients with pneumonia of unknown cause (1). After investigating the possible etiologic agent involved, on January 7, 2020, Chinese scientists isolated a new type of coronavirus from an individual and, therefore, were able to sequence its genome (2).

The SARS-CoV-2 or 2019-nCoV virus is the causative agent of the clinical syndrome known as COVID-19 (Coronavirus 19 disease) (3). Although SARS-CoV-2 belongs to the same gender as the viruses responsible for Severe Acute Respiratory Syndrome (SARS) and Middle East Respiratory Syndrome (MERS), the new coronavirus appears to be related to mild infections disorders but with a high rate of transmissibility (3–5). Considering the high levels of transmission, on March 11, the World Health Organization (WHO) characterized COVID-19 as a pandemic due to the rapid spread across countries, such as Italy, Spain and, later, United States, that currently has the highest number of cases of the novel coronavirus disease (6).

In Brazil, on February 26, 2020, the first case of COVID-19 was confirmed in the state of São Paulo and the first death on March 17, in the same state. During the months of April, May and June, the number of cases and deaths increased exponentially and, then, on June 20, 2020, Brazil was the second country in the world with the highest number of confirmed cases, more than 1 million, and also the second country with the most confirmed deaths, about 50 thousand (6).

In this context, the state of Minas Gerais, the second most populous state in the country, initially stood out for presenting an apparently controlled situation. While neighboring states in the southeastern region accumulated more than 300,000 cases and almost 22,000 deaths by COVID-19, Minas Gerais was one of the states with the least confirmed cases, approximately 27,000 (6). However, recent researches pointed to a possible underreporting scenario owing to the unprecedented increase in deaths from causes clinically similar to COVID-19, including SARS, respiratory failure and pneumonia, and due to the low number of tests performed by the state in comparison with the others, according to data obtained from the Minas Gerais Department of Health (7).

From this perspective, the high number of cases in neighboring states and underreporting in Minas Gerais rendered the region extremely susceptible to the increase in the number of cases of COVID-19, with the occurrence and diffusion of new cases. In this situation, delimiting and defining the main regions of vulnerability in the state is essential to guide the population, managers, public policies and government healthcare workforces. The concept of vulnerability, in this case, considers aspects that can measure whether the resources designated to the protection of people are available or in need. Thus, a complex set of indicators may determine a higher or lower vulnerability (8, 9).

In this work, the indicators, selected based on the literature to identify the vulnerability of the cities of Minas Gerais, describe physical, social and individual characteristics that enable to assess and qualify the regions with greater difficulty in managing the pandemic. These indicators, divided into demographic, social, economic, health infrastructure, population at risk and epidemiological, are directly related to the increase in illness and death due to the life-threatening condition.

Therefore, the aim of this study was to identify areas of higher vulnerability in the state of Minas Gerais and, from there, assess the region in a segmented and directed way (mesoregions of Minas Gerais) in order to contribute to the elaboration of public policies for prevention and combat of COVID-19.

## Methods

### Study Design

This is an epidemiological study to assess vulnerability based on Multi-Criteria Decision Analysis (MCDA) (10). The indicators were selected to allow the assessment of the state of Minas Gerais according to the vulnerability to COVID-19 at the municipal level.

### Study Area

The study assesses the state of Minas Gerais (MG), one of the 27 federative units in Brazil, located in the southeastern region of the country, and characterized as the second most populous state and the fourth with the largest territorial extension. It borders the states of São Paulo (south and southwest), Mato Grosso do Sul (west), Goiás (northwest), Bahia (north and northeast), Espírito Santo (east), and Rio de Janeiro (southeast). According to data provided by the Brazilian Institute of Geography and Statistics (IBGE), MG has an estimated population of 21,168,791, demographic density of 33.41 inhabitants per km^2^ (11, 12) in a territory of 586,521,123 km^2^, which corresponds, approximately, to the sum of the territorial areas of Spain and Portugal. The population is predominantly urban, 85.3% (12), composed of 22.3% of people from 0 to 15 years old, 69.3% from 15 to 64 years old and 8.1% above 65 years old. MG is the state with the third highest gross domestic product (GDP) in Brazil and has Human Development Index (HDI) of 0.731 (11, 12).

**Figure 1:**
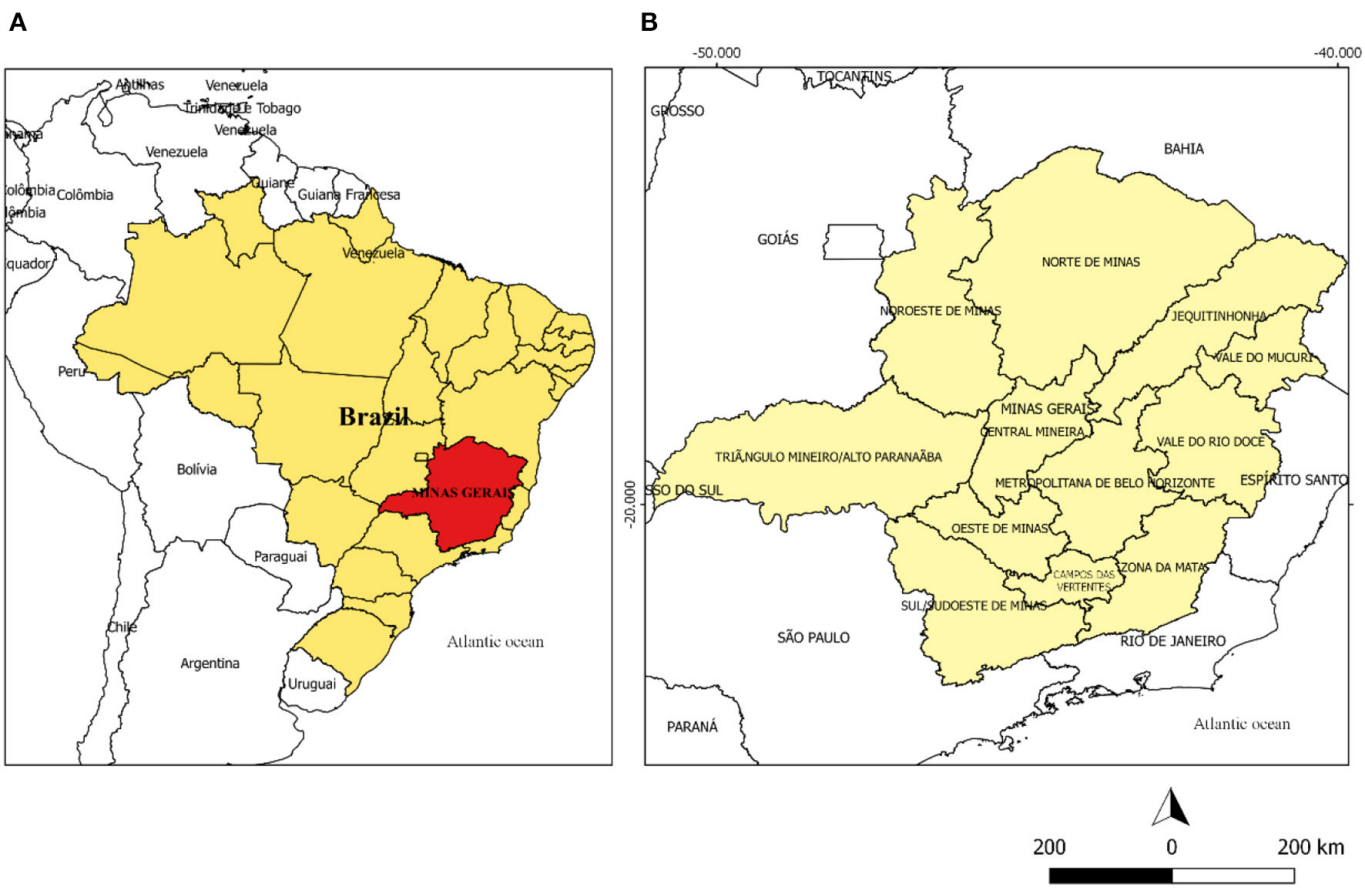
**(A)** Map of Brazil highlighting the state of Minas Gerais in red. **(B)** Map of the state of Minas Gerais divided into its mesoregions.

The state of MG, whose capital is located in the municipality of Belo Horizonte, has 853 municipalities distributed in 12 mesoregions: Noroeste de Minas, Norte de Minas, Jequitinhonha, Vale do Mucuri, Triângulo Mineiro and Alto Paranaíba, Central Mineira, Metropolitana de Belo Horizonte, Vale do Rio Doce, Oeste de Minas, Sul e Sudoeste de Minas, Campos das Vertentes, and Zona da Mata. Created in 1990 by IBGE, these mesoregions are guided by regional particularities related to social and administrative processes (13) (Figure 1).

### Data Analysis

The indicators used in this analysis were categorized into: demographic, social, economic, health infrastructure, population at risk, and epidemiological. In the process of selection of the indicators, articles that describe or analyze factors that could interfere directly in the increase of damage caused by COVID-19 in the population were selected based on a literature search in Scielo and PubMed databases. Then, among 18 articles selected (14–31), 23 indicators were identified, categorized and used in this paper. Other indicators that could potentially have some relation to the disease and death caused by coronavirus infection were not used because the official data available were limited at the municipalities level.

After selecting the indicators, these variables were collected from the databases of the Brazilian Institute of Geography and Statistics (“IBGE”) (https://www.ibge.gov.br/), Atlas of Human Development of Brazil 2013 (http://hdr.undp.org/en/content/new-atlas-human-development-brazil), from the National Supplementary Health Agency (NSHA) (http://www.ans.gov.br/), Mortality Information System (MIS) (http://www2.datasus.gov.br/DATASUS/index.php?area=060701), the National Registry of Health Establishments (NRHE) (http://www2.datasus.gov.br/DATASUS/index.php?area=0204&id=6906) and from the State Secretariat of Health of Minas Gerais (https://www.saude.mg.gov.br/). Then, 23 indicators remained, grouped according to categories (Table 1). The tabulated absolute data were adjusted to the respective values in percentage, incidence or mortality per 1,000 inhabitants, using information from the population projection of Minas Gerais for the year 2019 (11).

**Table 1:** Indicators by category (demographic, social, economic, health infrastructure, population at risk, epidemiological) used in the Multi-criteria Decision Analysis (MCDA) to assess the vulnerability of the municipalities of Minas Gerais to COVID-19.

**Category**	**Indicators**	**Description**	**Application in the context of COVID-19**	**Source**	**References**
Demographic	Percentage of the population living in an urban area	Percentage of the population residing in a situation of urban domicile in the municipalities of Minas Gerais.	There is a correlation between the increase in population density, proportion of built area, industrial concentration and other parameters associated with urbanization and increased morbidity by COVID-19. Additionally, air pollution, which is prevalent in locations with high rates of urbanization, contributes to the probability of infections.	2010 IBGE population census	(14, 15)
	Demographic density	Demographic density of the territorial unit (Inhabitant per square kilometer)	There is evidence that population density affects the number of COVID-19 daily cases.	2010 IBGE population census	(14, 16, 17)
Social	Percentage of inadequate sanitation	Households without basic sanitation condition, that is, they were not connected to the general water supply network, to sanitary sewage and had no access to garbage collection.	The virus that causes COVID-19 has already been detected in sewage samples in several countries and in the feces of infected patients, hence demonstrating the need for proper waste treatment.	2010 IBGE population census	(15, 18, 19)
	Illiteracy percentage	Rate of people aged 15 and over who cannot read or write	Important factor of social vulnerability, especially considering that one of the bases for combating the disease is information. Studies also indicate a higher prevalence of certain comorbidities in people with low levels of education.	2010 IBGE population census	(20, 21)
	Gini index	It measures the degree of inequality that exists in the distribution of individuals according to per capita household income.	Studies indicate that the Gini index can be extremely useful to measure the exposure-disease relationship.	Atlas of human development in Brazil 2010 (http://hdr.undp.org/en/content/new-atlas-human-development-brazil)	(22, 23)
	Municipal human development index	Geometric mean of the indexes related to income (per capita income indicator), Education (geometric average of the school attendance sub-index, with 2/3 weight, and of the schooling sub-index, with 1/3 weight) and Longevity (obtained through the life expectancy at birth), with equal weights.	The HDI can allow the assessment of social vulnerability by measuring the level of development of each region from three essential factors for quality of life.	Atlas of human development in Brazil 2010 (http://hdr.undp.org/en/content/new-atlas-humandevelopment-brazil)	(24)
Economics	Percentage of the population with per capita monthly income of up to 70 reais (BRL) (equivalent to US$ 13)	Population considered extremely poor.	Poverty and unemployment, characteristics of a population with such a low monthly income, are social determinants directly related to higher mortality caused by COVID-19.	2010 IBGE population census	(17)
	Percentage of the population with health insurance	Percentage of people by municipality who have access to health insurance.	Considering the need to treat more severe cases in Intensive Care Units (ICU) and the low availability of beds due to high demand, access to a private health network becomes an important indicator of less vulnerability.	National supplementary health agency TabNet DataSUS (March/2020)	(25, 26)
	Gross domestic product per capita	Proportion between the wealth produced by a municipality and its number of inhabitants.	The concentration of financial resources facilitates the promotion of measures to contain the pandemic, such as increasing the number of tests in the population.	2010 IBGE population census	(19)
Health infrastructure	Number of beds per 1,000 inhabitants	Proportion of the number of hospitalization beds by municipality.	Due to the pandemic, a great increase in the demand for health services is expected, thus it is essential to identify the most vulnerable regions and optimize the use of services and dimension resources that will be necessary to strengthen the response capacity of the health system regionally and locally.	TabNet DataSUS - Brazilian national registry of health facilities (NRHE) - Physical resources - 2019	(25, 27)
	Number of respirators per 1,000 inhabitants	Proportion of the number of respirators by municipality.			
	Number of doctors per 1,000 inhabitants	Proportion of the number of doctors by municipality.		TabNet DataSUS - Brazilian national registry of health establishments (NRHE) - Human resources - 2019	
	Number of nurses per 1,000 inhabitants	Proportion of the number of nurses by municipality.			
	Number of rapid tests per 1,000 inhabitants	Proportion of the number of rapid tests for COVID-19 performed by municipality.	Although total population testing is impractical, a well-designed program is essential to determine the prevalence of COVID-19 in the general society, in specific subgroups (including healthcare workers) and at-risk groups.	Data provided by the state health department of Minas Gerais on 06/22/2020	(28)
	Number of molecular tests (RT-PCR) per 1,000 inhabitants	Proportion of the number of molecular tests (RT-PCR) performed by municipality.			
Population at risk	Percentage of the population aged 60 or over	Percentage of the resident population in the municipalities of Minas Gerais aged 60 or over.	According to the World Health Organization, the mortality rate caused by COVID-19 increases with older age, with higher mortality among people over 80 years old.	2010 IBGE population census	(29)
	Mortality from diseases of the respiratory system per 1,000 inhabitants	Deaths per residence - Chapter ICD-10: X. Diseases of the respiratory system in 2018.	Cancer, hypertension, diabetes, Chronic Obstructive Pulmonary Disease (COPD), heart and cerebrovascular diseases are major risk factors for patients with COVID-19. Thus, municipalities with numerous cases of these life-threatening conditions become more vulnerable.	TabNet DataSUS mortality information system - MIS - 2018	(29–31)
	Mortality from diabetes per 1,000 inhabitants	Deaths per residence - Chapter ICD-10: IV. Diabetes (E10–E14) in 2018.			
	Mortality from neoplasms per 1,000 inhabitants	Deaths per residence - Chapter ICD-10: II. Neoplasms (tumors) in 2018.			
	Mortality from diseases of the circulatory system per 1,000 inhabitants	Deaths per residence - Chapter ICD-10: IX. Circulatory system diseases in 2018.			
Epidemiological	Incidence of COVID-19	Proportion between new cases of COVID-19 of a municipality and its population.	These occurrence measures are essential to compose an overview of COVID-19 in the municipalities of Minas Gerais, in addition to informing the evolution of the infectious illness in the state, a fact that would not be achieved only with the exposure of the absolute data of cases and deaths from the disease.	Epidemiological bulletin of the secretary of health of Minas Gerais on 06/22/2020	(31)
	Mortality of COVID-19	Number of COVID-19 deaths per 1,000 inhabitants.			
	Lethality of COVID-19	Proportion between the number of deaths caused by COVID-19 and the population affected.			

### Statistical Analysis

The vulnerability of the municipalities of Minas Gerais to COVID-19 was evaluated by a complex set of factors, herein represented by the indicators (32). For the joint analysis of these factors, an instrument known as Multi-criteria Decision Analysis (MCDA) was applied through the Program to Support Decision Making Based on Indicators (PRADIN) software, in order to represent the vulnerability of these municipalities (33).

The use of this approach in Health Surveillance is recommended by the Ministry of Health of Brazil as an analysis methodology for the management process of the Brazilian Unified Health System (SUS) (34). Importantly, the method has already been used to classify areas of vulnerability for *Trypanosoma cruzi* (35) and to map the vulnerability to hantavirus infections in Brazil (36). Likewise, in the United States, the methodology was used to analyze the risk of Chagas disease in Texas (37).

In order to facilitate the understanding of the statistical analysis performed, we divided the session into: **Decision-making**, seeking to explain the tools used in the analysis and choice of indicators; **Simulations**, explaining what the simulations are and how they work; **Categorization**, showing how we organize the indicators in the study; **Weights**, explaining the distribution of the weights of the indicators; **Organization of results**, explaining how the data were grouped and placed on thematic maps.

### Decision-Making

Multi-criteria Decision Analysis (MCDA) can be conceptualized as a set of strategies to assist in making decisions about a complex problem. MCDA uses and evaluates several criteria and perspectives aimed at identifying priorities, better health management and planning solutions (10). The technique allows the decision to occur based on what the decision-makers consider to be relevant to the situation (10). Thus, applying the concept of this methodology (MCDA) to the present study, different indicators selected by the authors were evaluated to identify the most vulnerable areas of Minas Gerais to COVID-19 and, therefore, priorities for public policies to combat the pandemic.

**Figure 2:**
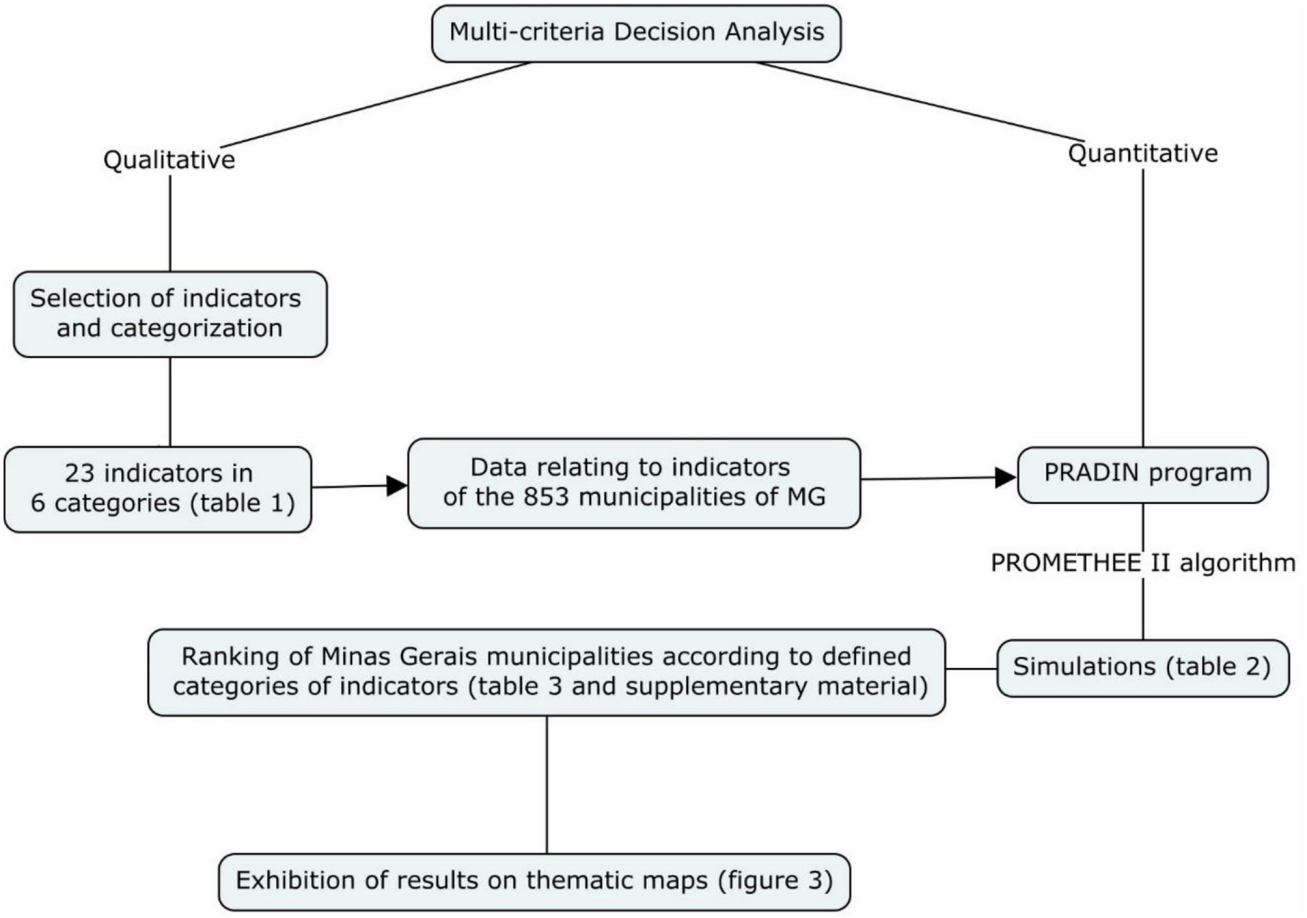
Flowchart with step-by-step description of the methodology.

MCDA is a qualitative and quantitative process (Figure 2). The qualitative phase corresponds to the choice of factors related to the analyzed problem, the indicators (Table 1). In turn, the quantitative phase refers to the techniques that are employed in the search for a multi-criteria solution, which have already been described and analyzed by the literature (10, 38). The choice of the technique depends on how the problem presents itself and how it relates to the qualitatively selected factors (10, 38).

The present work used the Preference Ranking Organization Method for Enrichment of Evaluations, the PROMETHEE technique, specifically variant 2, because this is an over-classification method that allowed the municipalities to be structured hierarchically, based on indicators related to the complex problem, in order to determine priority levels (38). The technique allows simulations to be performed (Table 2) by comparing the municipalities for each defined indicator, according to functions capable of defining the overcoming of one city in relation to another.

**Table 2:** Simulations performed by category of indicators (1–6) and the general simulation (7), gathering all the indicators simultaneously.

**Indicators**	**Simulations**						
	**1st**	**2nd**	**3rd**	**4th**	**5th**	**6th**	**7th**
**Demographic**							
Population percentage living in urban area	X						X
Demographic density	X						X
**Social**							
Percentage of inadequate sanitation		X					X
Human development index		X					X
Illiteracy percentage		X					X
Gini index		X					X
**Economic**							
Population percentage with monthly income higher than 70 reais (equivalent to US$ 13)			X				X
Population percentage with health insurance			X				X
Gross domestic product			X				X
**Healthcare infrastructure**							
Number of respirators by 1,000 inhabitants				X			X
Number of beds by 1,000 inhabitants				X			X
Number of nurses by 1,000 inhabitants				X			X
Number of doctors by 1,000 inhabitants				X			X
Number of rapid tests by 1,000 inhabitants				X			X
Number of molecular tests (RT-PCR) by 1,000 inhabitants				X			X
**Population at risk**							
Mortality from respiratory diseases by 1,000 inhabitants					X		X
Mortality from cardiovascular diseases by 1,000 inhabitants					X		X
Mortality from neoplasm by 1,000 inhabitants					X		X
Mortality from diabetes by 1,000 inhabitants					X		X
Population percentage with 60 years or more					X		X
**Epidemiological**							
COVID-19 incidence by 1,000 inhabitants						X	X
COVID-19 mortality by 1,000 inhabitants						X	X
COVID-19 lethality						X	X
All indicators (general)	X	X	X	X	X	X	X

The PROMETHEE II method was computationally implemented to the Program to Support Decision Making Based on Indicators (PRADIN) in order to facilitate its operationalization. Therefore, using the functions of the PROMETHEE algorithm, associated with PRADIN, simulations can be performed for the selected indicators. The values (0–1) received by each city in the simulations are known as multi-criteria indicators in the app, and from there, the hierarchy for vulnerability classification occurs.

### Simulations

After collecting the data related to the chosen indicators, these data were inserted in the program, PRADIN, to be compared and hierarchized. In the present work, it was called simulation every time the program performs this process of comparison and hierarchization of the chosen indicators. Thus, these simulations indicate how the data was organized in the app.

### Categorization

To facilitate the use of this analysis by health managers, the study divided the indicators into categories (demographic, social, economic, health infrastructure, population at risk, epidemiological) and carried out separate simulations with indicators belonging to each one. Thereby, the vulnerability can be analyzed in different perspectives, allowing segmented interventions based on the categories, which are usefull according to the interest of health managers.

Therefore, the simulations occurred in a segmented manner, according to groupings, evaluating the group of indicators by the pre-defined categories (Table 2). Posteriorly, an analysis was undertaken from a joint simulation of all multi-criteria indicators to establish the vulnerability scenario for Minas Gerais at the municipal level for COVID-19. This general simulation enabled a more complete view of the pandemic scenario at Minas Gerais. Six simulations were conducted, with the groupings of indicators and a general simulation, covering all 23 indicators, therefore totaling seven simulations (Table 2).

For the development of the MCDA, each city of Minas Gerais was included in a spreadsheet and received a set of data related to the indicators (Table 1), which were analyzed by the PRADIN program according to the total number of municipalities in order to classify them according to their vulnerability (worst indicators reflect greater vulnerability).

### Weights

The weights given to the indicators (0–10) in the MCDA were established by the decision-makers. In this work, all indicators received the same weight in the hierarchy process, since no evidence was found on which factor influences COVID-19's illness and death processes to a higher or lower extent. For the authors of the present study, giving different weights to the indicators is inconsistent in view of the complexity of the pandemic scenario experienced in Brazil, a country full of structural problems, so that any type of assumption about the greater or lesser importance of the indicators without accurate legitimation by the scientific literature could invalidate the results obtained.

### Results Organization

After the analysis of the indicators by MCDA, each simulation was classified into quintiles, according to the multi-criteria indicator (MCI) and divided into groups of vulnerability according to municipalities, mesoregions and population size, classified in an ascending order. Groups 1 and 2 were composed by the municipalities with the least vulnerability, group 3 with moderate vulnerability and groups 4 and 5 were those with the greatest vulnerability.

After the classification and definition of the groups, the codes of the municipalities and the program Tabwin 32 (http://www2.datasus.gov.br/DATASUS/index) were used to construct the vulnerability maps for COVID-2019. In thematic maps, the darker colors of the municipalities represent greater vulnerability, while the lightest colors indicate lower vulnerability. For the organization and evaluation of the collected data, the regionalization of the municipalities of Minas Gerais in Mesoregions was used (Figure 1).

To compose the data analysis, the concept of population size of each municipality was also used (39), categorizing in small municipalities those with up to 25 thousand inhabitants, in medium-sized municipalities those with population between 25 and 100 thousand inhabitants and in large municipalities those with more than 100 thousand inhabitants.

Due to the advantages of the technique, other studies used this tool to assist in decision making considering disease surveillance programs (35–37, 40–42). Interestingly, a recent study carried out in India performed an analysis very similar to this study, in which researchers mapped the vulnerability of India to COVID-19 (41). Given the importance of the method and its increasingly recurrent use, a systematic review was conducted to assess and synthesize articles that used multi-criteria analysis for decision making in health area (42). This review highlighted the methodological variety that can be used to construct MCDA, with the collaboration of both literature and decision makers and experts in the process of evaluating the best criteria and discussing the results for evaluation of the decision (42).

## Results

**Figure 3:**
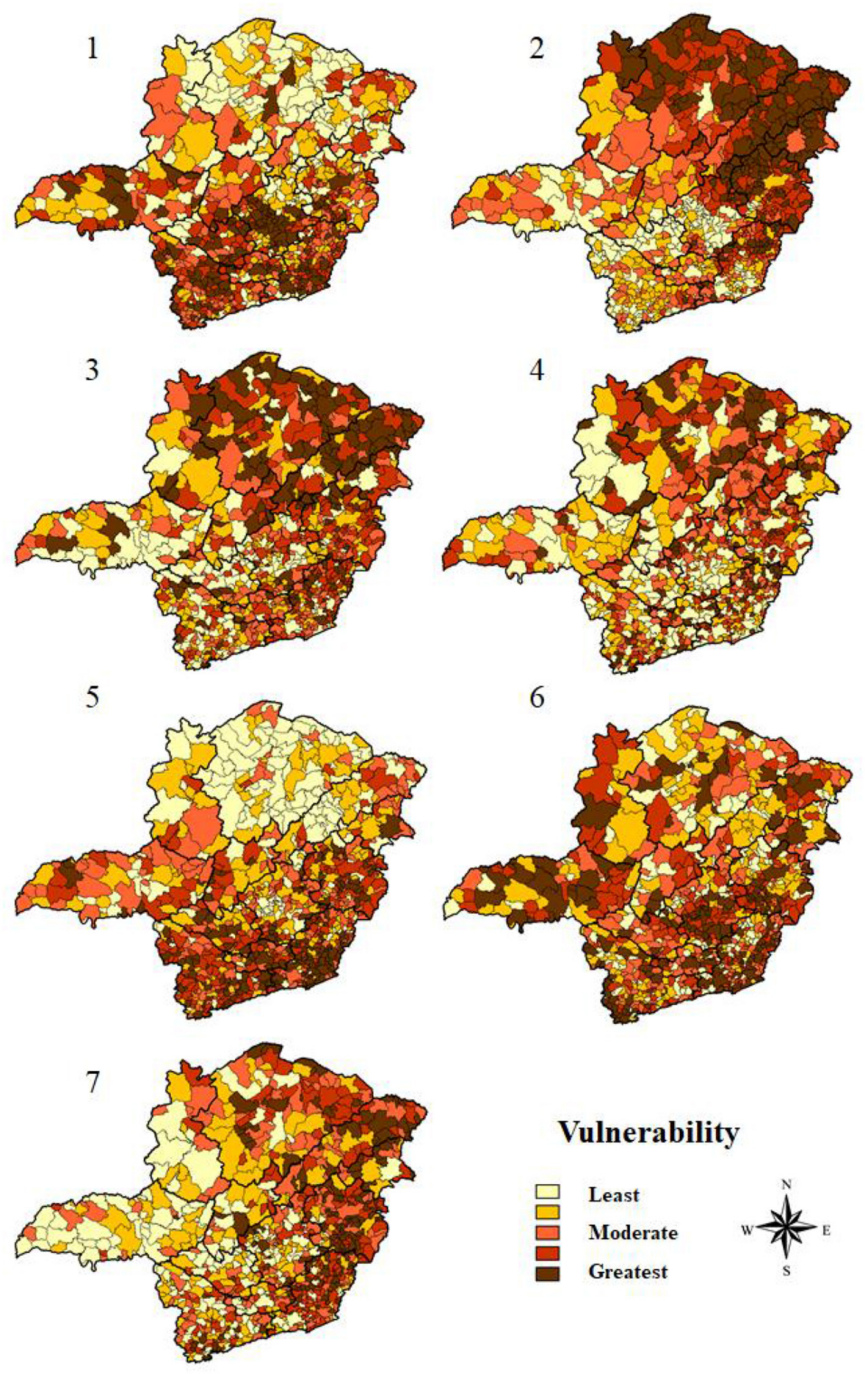
Thematic maps of the vulnerability simulations of the municipalities of Minas Gerais for COVID-19, based on the multi-criteria decision analysis.

The results obtained by the multi-criteria analysis of the groupings of indicators are depicted in Figure 3, according to the analysis of the 853 municipalities in Minas Gerais (Supplementary Table 1). Table 3 shows the mesoregions of the most and least vulnerable municipalities in Minas Gerais for COVID-19, in accordance with the grouping of indicators used in the multi-criteria analysis of decision.

**Table 3:** Mesoregions of municipalities and groups of vulnerability (1 and 2 representing lower vulnerability, 3 moderate vulnerability and 4 and 5 greater vulnerability) for COVID-19 in the state of Minas Gerais, according to indicators used in the multi-criteria decision analysis.

**Mesoregions and groups**		**Indicators (%)**						
		**Demographic**	**Social**	**Economic**	**Healthcare infrastructure**	**Population at risk**	**Epidemiological**	**General**
Campo das vertentes	1	8.3	19.4	11.1	22.2	5.6	16.7	25.0
	2	25.0	33.3	27.8	25.0	16.7	13.9	22.2
	3	22.2	33.3	41.7	19.4	16.7	30.6	25.0
	4	19.4	13.9	11.1	13.9	27.8	19.4	19.4
	5	25.0	0.0	8.3	19.4	33.3	19.4	8.3
Central mineira	1	23.3	16.7	23.3	30.0	20.0	33.3	40.0
	2	26.7	30.0	20.0	20.0	20.0	13.3	23.3
	3	23.3	33.3	16.7	20.0	20.0	16.7	13.3
	4	20.0	16.7	26.7	20.0	26.7	23.3	16.7
	5	6.7	3.3	13.3	10.0	13.3	13.3	6.7
Jequitinhonha	1	37.3	0.0	3.9	9.8	45.1	23.5	0.0
	2	33.3	2.0	13.7	17.6	21.6	33.3	17.6
	3	19.6	7.8	11.8	21.6	21.6	17.6	23.5
	4	9.8	31.4	13.7	29.4	11.8	15.7	21.6
	5	0.0	58.8	56.9	21.6	0.0	9.8	37.3
Metropolitana de belo horizonte	1	17.1	42.9	25.7	28.6	28.6	15.2	27.6
	2	21.9	15.2	25.7	20.0	25.7	13.3	25.7
	3	7.6	16.2	20.0	26.7	19.0	17.1	21.9
	4	10.5	12.4	17.1	12.4	12.4	27.6	13.3
	5	42.9	13.3	11.4	12.4	14.3	26.7	11.4
Noroeste de minas	1	42.1	10.5	5.3	26.3	31.6	10.5	31.6
	2	26.3	15.8	31.6	15.8	42.1	36.8	26.3
	3	31.6	42.1	26.3	0.0	10.5	5.3	31.6
	4	0.0	15.8	15.8	26.3	15.8	36.8	10.5
	5	0.0	15.8	21.1	31.6	0.0	10.5	0.0
Norte de minas	1	57.3	1.1	11.2	7.9	61.8	30.3	5.6
	2	23.6	2.2	11.2	11.2	25.8	29.2	19.1
	3	7.9	11.2	11.2	15.7	10.1	20.2	25.8
	4	6.7	40.4	19.1	23.6	2.2	6.7	28.1
	5	4.5	44.9	47.2	41.6	0.0	13.5	21.3
Oeste de minas	1	15.9	50.0	40.9	31.8	6.8	13.6	36.4
	2	9.1	25.0	20.5	20.5	27.3	20.5	22.7
	3	13.6	25.0	22.7	25.0	20.5	38.6	22.7
	4	31.8	0.0	9.1	6.8	29.5	15.9	15.9
	5	29.5	0.0	6.8	15.9	15.9	11.4	2.3
Sul/sudoeste de minas	1	6.2	32.2	26.7	23.3	6.8	18.5	26.0
	2	11.0	43.8	26.7	24.7	11.6	23.3	28.1
	3	27.4	19.9	26.0	16.4	19.9	19.9	17.1
	4	28.8	3.4	12.3	16.4	34.2	16.4	17.1
	5	26.7	0.7	8.2	19.2	27.4	21.9	11.6
Triângulo mineiro/alto paranaíba	1	19.7	28.8	39.4	25.8	22.7	13.6	57.6
	2	19.7	30.3	28.8	31.8	24.2	16.7	24.2
	3	19.7	34.8	16.7	19.7	31.8	12.1	13.6
	4	24.2	6.1	7.6	19.7	13.6	30.3	3.0
	5	16.7	0.0	7.6	3.0	7.6	27.3	1.5
Vale do mucuri	1	43.5	0.0	17.4	4.3	13.0	26.1	4.3
	2	17.4	0.0	8.7	17.4	30.4	21.7	17.4
	3	17.4	8.7	13.0	17.4	39.1	30.4	21.7
	4	21.7	13.0	17.4	26.1	13.0	8.7	8.7
	5	0.0	78.3	43.5	34.8	4.3	13.0	47.8
Vale do rio doce	1	16.7	4.9	9.8	11.8	7.8	19.6	3.9
	2	26.5	5.9	19.6	12.7	20.6	22.5	6.9
	3	18.6	7.8	11.8	25.5	20.6	15.7	17.6
	4	24.5	42.2	36.3	28.4	26.5	24.5	27.5
	5	13.7	39.2	22.5	21.6	24.5	17.6	44.1
Zona da mata	1	6.3	12.7	14.8	19.0	7.0	21.1	9.2
	2	16.9	19.0	11.3	21.1	12.0	11.3	14.1
	3	30.3	26.1	24.6	19.0	19.7	22.5	19.0
	4	23.9	26.8	32.4	21.8	19.0	20.4	30.3
	5	22.5	15.5	16.9	19.0	42.3	24.6	27.5

From the analysis of demographic indicators, the results indicated that the Norte de Minas mesoregion is the least vulnerable (Figure 3-1). Accordingly, this mesoregion had 80.9% of the municipalities with the least vulnerability, groups 1 and 2, and 9 municipalities among the 10 least vulnerable in the state for these indicators. Contrarily, the Metropolitana de Belo Horizonte had 53.4% of its municipalities classified in groups 4 and 5, that is, with greater vulnerability (Table 3). Thus, 7 of the 10 most vulnerable municipalities in Minas Gerais were located in this mesoregion. The findings also revealed that 97.0% of the municipalities categorized as large ones were included in group 5 of vulnerability (Table 4).

**Table 4:** Results of the multi-criteria decision analysis according to the population size of municipalities in the state of Minas Gerais and their vulnerability classification, in accordance with the grouping of indicators used in the multi-criteria decision analysis for COVID-19.

**Cities by population size**	**Total of cities**	**Groups**	**Indicators (%)**						
			**Demographic**	**Social**	**Economic**	**Healthcare infrastructure**	**Population at risk**	**Epidemiological**	**General**
Small size (until 25 thousand inhabitants)	705	1	23.3	12.9	16.2	11.1	18.6	24.3	17.0
		2	22.7	19.9	20.0	20.0	18.9	21.7	18.2
		3	22.8	22.3	21.7	21.3	20.6	21.8	21.1
		4	20.7	22.8	21.1	23.5	20.0	20.1	21.1
		5	10.5	22.1	21.0	24.1	22.0	12.1	22.6
Medium size (25–100 thousand inhabitants)	115	1	6.1	45.2	32.2	56.5	26.1	0.0	31.3
		2	9.6	23.5	20.0	24.3	26.1	15.7	27.8
		3	8.7	11.3	13.9	13.9	16.5	14.8	14.8
		4	20.9	8.7	17.4	4.3	20.9	24.3	18.3
		5	54.8	11.3	16.5	0.9	10.4	45.2	7.8
Large size (more than 100 thousand inhabitants)	33	1	0.0	84.8	54.5	78.8	30.3	0.0	45.5
		2	0.0	12.1	21.2	6.1	24.2	0.0	33.3
		3	0.0	3.0	6.1	15.2	21.2	0.0	15.2
		4	3.0	0.0	6.1	0.0	18.2	3.0	3.0
		5	97.0	0.0	12.1	0.0	6.1	97.0	3.0

From the analysis of social indicators, the results showed that the mesoregions Oeste de Minas and Metropolitana de Belo Horizonte occupy, together, the position of the least vulnerable. Both have 63.0% of their municipalities integrating groups 1 and 2. In contrast, Norte de Minas, Vale do Rio Doce and Vale do Mucuri stand out as the most vulnerable (Figure 3-2). While the first two mesoregions have, respectively, 85.3 and 81.4% of their municipalities distributed between groups 4 and 5, the Vale do Mucuri mesoregion exhibits 91.3% of its municipalities integrating the most vulnerable groups, where 4 of the 10 most vulnerable cities are located in this mesoregion (Table 3). Besides, 84.9% large and 45.2% medium-sized municipalities were among the least vulnerable in relation to social indicators (Table 4).

Regarding economic indicators, Triângulo Mineiro/Alto Paranaíba mesoregion was the least vulnerable region in the state, with 68.2% of the municipalities classified in groups 1 and 2 (Figure 3-3). The Jequitinhonha and Norte de Minas mesoregions had 56.9% and 47.2% of their municipalities categorized in group 5, respectively. Importantly, 6 most vulnerable municipalities in the state belong to these regions (Table 3). Further, within this analysis category, 52.2 and 75.7% of medium-sized and large cities, respectively, are part of the groups 1 and 2 and, therefore, have lower vulnerability (Table 4).

When analyzing the indicators that assess health infrastructure, the results reported that Oeste de Minas and Central Mineira mesoregions are less vulnerable, with 31.8 and 30.0% of the municipalities in group 1, respectively (Table 3). Norte de Minas and Vale de Mucuri were among the most vulnerable areas, with 41.6 and 34.8% of the municipalities in group 5, respectively (Figure 3-4) (Table 3). In this regard, medium-sized and large municipalities with, respectively, 80.8 and 84.9% in groups 1 and 2, have lower vulnerability (Table 4).

Considering the estimation of population at risk, the findings showed that the mesoregion of Norte de Minas was the least vulnerable, with 61.8% of its municipalities classified in group 1. Notwithstanding that, Zona da Mata mesoregion proved to be the most vulnerable, with 42.3% of its municipalities being part of group 5 (Table 3) (Figure 3-5). Furthermore, 22.0% of the municipalities classified as small-sized were part of group 5 (Table 4).

Based on epidemiological indicators, the Norte de Minas and Jequitinhonha mesoregions stood out due to their lower vulnerability with, respectively, 59.6 and 56.9% of the municipalities classified in groups 1 and 2. In turn, Metropolitana de Belo Horizonte and Triângulo Mineiro/Alto Paranaíba revealed higher vulnerability, with 54.3% and 57.6% of the municipalities classified in groups 4 and 5, respectively (Table 3) (Figure 3-6). However, 7 of the 10 most vulnerable municipalities in the state are located in the mesoregion of Zona da Mata. Moreover, the findings reported that 45.2% of medium-sized municipalities and 97.0% of large municipalities were classified in group 5 (Table 4).

The analysis of all indicators jointly demonstrated that Triângulo Mineiro/Alto Paranaíba is the least vulnerable mesoregion (Table 3) (Figure 3-7). With 57.6% of its municipalities classified in group 1, this region contains 5 of the 10 least vulnerable municipalities. It is noteworthy to highlight the positions of Vale do Mucuri and Vale do Rio Doce. While the first mesoregion has the highest rate of municipalities occupying group 5 (47.8%), Vale do Rio Doce comprises 5 of the 10 most vulnerable municipalities, with 44.1% of its municipalities integrating group 5 (Table 3) (Figure 3-7). Additionally, the general analysis revealed that small municipalities are among the most vulnerable, with 43.7% of their representatives divided between groups 4 and 5 (Table 4).

## Discussion

The use of MCDA to generate these results was based on the perspective that the health-illness-care process depends on several factors determined by the individuals' living conditions. Thus, exploring the structure and spatial dynamics of the population is essential for the characterization of health situations to plan actions and allocate resources (15, 43). From this perspective, in order to draw a panorama of reality, the use of indicators becomes an important instrument to measure it in a succinct, objective, quick and efficient way, aiming to support an intervention (43).

In the context of the COVID-19 pandemic, it is emphasized that the novel coronavirus infection has the potential to affect everyone in society, however in a heterogeneous way (44), therefore requiring identification of areas of vulnerability. Although various strategies for mitigating the rate of disease transmission are recommended for the entire community, it is pivotal to examine areas based on their unique characteristics, including demographic variation, economic aspects, health conditions of population and characteristics of the health system, in order to produce improved and targeted interventions (45). Thus, in a state with a projection of more than 20 million inhabitants in 2019 and more than 580 thousand km^2^ of area (IBGE), the peculiarities of each region become even more accentuated.

In this scenario, Minas Gerais occupied, in 2012, the 9th position in the national urbanization ranking (24), but its territorial vastness causes important internal inequalities regarding the urbanization rates of the municipalities, a relevant fact for vulnerability analysis of COVID-19 (46). Indeed, COVID-19 is closely related with high population density owing to the high degree of social interactions. In this sense, individuals living in urban areas are more likely to test positive for the disease when compared to individuals living in rural areas (47, 48). Thus, the lower vulnerability verified in Norte de Minas mesoregion is due to the much lower urbanization rates in comparison to the average state, in addition to the low demographic density and predominance of smaller cities (12). Furthermore, proving this strong relationship, it is important to mention the specificity of Montes Claros (Figure 3-1), a municipality in the Norte de Minas that differs in terms of higher rates of urbanization and, for this reason, exposed a similar profile to the municipalities located in the center-south portion of Minas Gerais, with high vulnerability, comparable to the Metropolitana de Belo Horizonte mesoregion (49).

According to the Atlas of Social Vulnerability in Brazilian Municipalities (50), by 2015, all thirty municipalities in the Southeast region classified as high social vulnerability were located in Minas Gerais. Further, considering the COVID-19 transmission scenario, locations with better education, sanitation and development indicators concentrate greater instructional and sanitary capacity to contain the spread of the virus (18). Thus, in the analysis of vulnerabilities, large and medium-sized municipalities, predominant in the central-southern portion of the state, demonstrated less risk, given that the best social indicators are verified in Oeste de Minas and Metropolitana de Belo Horizonte mesoregions. In contrast, the northern mesoregions, especially Vale do Mucuri, are the ones with the worst educational and housing conditions (50). The northern portion of the state herein proved to be more socially vulnerable to COVID-19, demanding public policies directed to improving these indicators to overcome the contagion.

In addition, from the analysis of economic indicators, the results indicated greater vulnerability in the northern region of Minas Gerais, mainly in Jequitinhonha and Norte de Minas, in contrast to Triângulo Mineiro/Alto Paranaíba and Metropolitana de Belo Horizonte. Remarkably, this information is relevant as, in the context of the pandemic, the politicians have focused on populations at risk considering mainly comorbidities and age (46). However, socioeconomic issues have been in the background, which may favor COVID-19 exposure and mortality. Additionally, economically disadvantaged people are more likely to live in accommodation with high number of people and less access to open areas, besides to having unstable occupations that do not allow remote office work (46). In this sense, the current prevention model based mainly on social isolation can be fragile and limited when applied to needy, isolated and low-educated populations (51). Therefore, poverty represents a hurdle to effective measures to contain the pandemic and must be taken into account in public policy decision making.

Regarding the health infrastructure indicators, a higher vulnerability was also found in the northern regions. This category is especially important when considering more severe cases of the disease, which require hospitalization in Intensive Care Units (ICU) (26). Inadequate health infrastructure directly influences the mortality rate caused by COVID-19. In this context, the Brazilian health regions with the highest mortality rates are located in places where the shortage of ICU beds and ventilators is more prevalent (27). Thus, the saturation of ICU and respirators resulting from the increasing demand becomes an aggravating factor for the COVID-19 pandemic and requires attention from managers (25). In this context, the construction of temporary hospitals, as has already been done in other parts of Brazil, may be an alternative.

Considering the number of tests for COVID-19, an important factor in determining the prevalence of infection/disease in the population (28), Brazil, as the vast majority of other developing countries, has a very modest number when compared to developed countries (52). Despite being the second country with the highest number of absolute deaths and the fifth with the highest number of deaths per million inhabitants, Brazil is only the 14th country testing patients, hence demonstrating a serious concern (53). The context of Minas Gerais is even more worrying, considering that the state has the third lowest number of tests per thousand inhabitants among the 26 states and 1 federative unit (6). This has failed to identify potential transmitters and directly influences the number of reported cases, which may be much lower than the actual number. Additionally, questions have been raised about possible underreporting of cases, which further aggravates the state's situation, thus making government intervention urgent (7).

The factors of comorbidities and age, which compose the population at risk indicators, were raised during the pandemic in order to draw a well-defined profile of people more susceptible to the complications of COVID-19. Both in Wuhan, China (54) and in the Italian states (55), respiratory and cardiac diseases, as well as neoplasms, diabetes and advanced age are considered factors for complications of the clinical condition and of higher mortality. In this sense, access to health infrastructure and education acts as an aggravation of diseases, and the region that is able to provide more appropriately these resources to the population ensures better conditions to prolong life (56). Importantly, a greater longevity is also accompanied by an increase in the elderly population, which is more affected by chronic diseases, and with regard to COVID-19, these diseases act as complicating factors of the clinical condition. Thus, as reported by this study, the worst social and health infrastructure indicators in the Norte de Minas may be associated with lower longevity, hence leading the northern portion of the State to have fewer people in the risk group (57). On the other hand, the Zona da Mata is better assisted, which leads its population to be longer-lived and, consequently, to have a higher number of people more susceptible to the health complications caused by COVID-19.

Another pivotal issue is that coping with the COVID-19 pandemic involves changes in the health system and also requires political decisions that affect the management of chronic non-communicable diseases, as well as patients' adherence to treatment, especially those from less favored social classes (58). Furthermore, the pandemic scenario increases patients' fear of seeking health services, which can increase mortality from events related to the chronic illness (59). Based on the identification of this regional vulnerability profile, it is possible to outline public policies that address the major diseases associated with the worsening of the clinical condition of patients with COVID-19, as well as specialized care for the elderly, markedly more affected by the condition.

With regard to epidemiological aspects, greater vulnerability was found in the metropolitan mesoregions of Metropolitana de Belo Horizonte, Triângulo Mineiro/Alto Paranaíba and Zona da Mata and, in a lesser extent, in the Norte de Minas and Vale do Mucuri. In this sense, the observance of the great impact of the mesoregions further south and southeast of the state becomes relevant when considering that these places are located on the border with the states of São Paulo and Rio de Janeiro, which concentrate the highest number of cases in Brazil. Besides that, the lower socioeconomic development in the north of Minas Gerais favors the scenario of underreporting of cases in the state, which can be associated with a large north-south discrepancy in the numbers found. Thus, social distance should be considered through reliable measures, including travel restrictions or even the institution of lockdown, which have proven effective in countries such as China, South Korea, Iran, Italy, France and the United States (60). In addition to these measures, others widely used worldwide must be promulgated with greater avidity in the most affected municipalities, including awareness about the use of personal protective equipment, social distance, closing schools and business buildings, quarantine, cleaning and disinfection and increase in the number of tests (19, 61–64).

Considering the joint analysis of all indicators, the lower vulnerability of Triângulo Mineiro and Alto Paranaíba was proven, with better social, economic, population at risk and health infrastructure indicators. Thus, the highest human development indexes, in addition to a diverse and historically integrated economy to the State of São Paulo, associated with a higher presence of young people and the concentration of hospital resources, integrate factors to reduce vulnerability to COVID- 19 (65, 66). Similarly, the findings also reported the greatest vulnerability in Vale do Mucuri, followed by Vale do Rio Doce. In these areas, the economy is fragile, basically composed of the primary sector. Education and sanitation indicators are remarkably low and there is a predominance of higher age groups (65). Additionally, the few existing hospital resources are concentrated especially in the municipalities of Teófilo Otoni and Governador Valadares, not reaching all the surrounding municipalities (66).

Besides to the evident differences revealed by the indicators between the mesoregions of Minas Gerais, data also exhibited differences between the municipalities grouped by population size, with emphasis on the numerical predominance of small municipalities. In this sense, the analysis performed in the present study is essential to better understand the possible particularities of these municipalities in the face of the pandemic, highlighting the need to formulate specific strategies and public policies according to the size of the population.

The high transmissibility of SARS-COV-2 in large urban centers with population agglomerations results in a rapid and exponential increase in the number of cases and deaths from the entry of the virus into the population (65). In fact, the rapid increase in the absolute number of cases requires the development of containment measures and, in some cases, when applied with due urgency, relative success is achieved. However, the pandemic is not restricted to large municipalities, but also reaches medium-sized and small areas, consolidating the internalization of the disease, which in Brazil reaches more than 90% of the municipalities (6, 67).

The smaller cities did not have large absolute number of cases and deaths compared to the others, but when investigating the proportion measures, including incidence, lethality and mortality, as performed by this work, it is clear that several small municipalities were in a serious situation. Thus, the exposed and disseminated absolute data of these cities do not cause as much impact on the population and public managers as they should, making containment measures, such as social distance, take time to be employed or adhered by the population and managers.

This scenario is particularly worrying for the small municipalities that were, for the most part, more vulnerable, especially in relation to health infrastructure and financial resources (68). A large portion of these ones reported few ICU beds, few or no respirators and a reduced number of health professionals, leading to a high lethality of the disease, since the basic care conditions of the most serious cases of COVID-19 are not guaranteed (45).

Based on the regionalization process defined by the Minas Gerais Health Regionalization Master Plan, these small municipalities should be assisted by medium and large-sized cities, enabling access to medium and high complexity services in severe cases of the disease (27). However, the reality of the state of Minas Gerais does not meet this proposal, as the assessment demonstrated several discontinuities and inequalities in all indicators of the state. Alarmingly, the small municipalities are isolated in the middle of the pandemic, without support from the medium-sized and large municipalities and without enough resources to improve their own health infrastructure (66, 68). Municipalities listed to offer this support face the overcrowding of beds and the lack of respirators (25).

Importantly, some indicators that could contribute to the mapping of the vulnerability of Minas Gerais to COVID-19 were not included due to the absence of data related to the municipalities, hence hindering the tabulation. The data included also had a difference in the dates on which they were made available, since some are only accessed by the 2010 demographic census conducted by IBGE. Another limitation is also related to the database available for consultation, since various indicators may be out of date, especially in small municipalities where the registration process does not occur or is not done properly, showing the presence of under-notification of cases. Nevertheless, it is important to use these data to evaluate the vulnerability considering the risk of worsening disease, since this article intends to measure the capacity of the cities to contain COVID-19 and its complications.

In addition, in the data tabulation process for MCDA, one of the steps consists in defining weights for the different data included, herein determining which ones would have different intensities of influence. However, the present study chose to keep all data with the same weight owing to the lack of evidence, showing which factors would have a higher or lower influence in the pandemic and the difficulty of stipulating the proportion of this influence. Therefore, the maps and the findings of this study should be used only as an instrument of guidance for public policies with other existing tools, and not as the only resource. Also, a segmented analysis may be performed by category of indicators in order to avoid possible differences in the influence of indicators in the compilation of the final result.

From the vulnerability analysis performed, it is clear that the demands of the municipalities of Minas Gerais in the context of COVID-19 are different, varying according to the region in which they are located and their population size. Thus, a public policy planned for the state will have totally different applicability and effectiveness depending on the region or municipality in question. Therefore, a more segmented analysis of the state should be conducted, as proposed by this work, in order to identify the particularities of each municipality and mesoregion in the search for interventions that have an effect in a faster and more practical way, as the context requires. In this scenario, measures are needed to contain the spread of the disease in the state as a whole, not just in the most economically important regions. For this, the problem of capillarity of the state related to social, economic and health indicators must be solved, so that everyone has a similar capacity to fight the pandemic. As a result, the state will not only benefit from combating the COVID-19 pandemic, but also from combating all inequalities that have been consolidated in Minas Gerais and directly affect the quality of life of the population in the less-assisted regions.

## Data Availability Statement

The datasets presented in this study can be found in online repositories. The names of the repository/repositories and accession number(s) can be found in the article/Supplementary Material.

## Author Contributions

SO contributed to the conception of the study. SO and IC contributed to the acquisition, analysis and interpretation of data, contributed to the statistical analysis, interpretation of data, and creation of table and figures. VA, KC, and JL participated in revising it critically for important intellectual content for discussion topic. All the authors co-wrote the paper and give final approval to the version to be submitted.

## Conflict of Interest

The authors declare that the research was conducted in the absence of any commercial or financial relationships that could be construed as a potential conflict of interest.
